# Utility of somatosensory evoked potentials in the assessment of response to IVIG in a long-lasting case of chronic immune sensory polyradiculopathy

**DOI:** 10.1186/s12883-017-0906-2

**Published:** 2017-07-01

**Authors:** Angelo Maurizio Clerici, Eduardo Nobile-Orazio, Marco Mauri, Federico Sergio Squellati, Giorgio Giovanni Bono

**Affiliations:** 1 Neurology Unit, Circolo & Macchi Foundation Hospital - Insubria University – DBSV, Viale L. Borri 57, 21100 Varese, Italy; 2 2nd Neurology, Humanitas Clinical and Research Institute, Department of Medical Biotechnology and Translational Medicine (BIOMETRA), Milan University, Rozzano, Milan, Italy

**Keywords:** Chronic immune sensory polyradiculopathy, Somatosensory evoked potentials, Chronic inflammatory demyelinating polyneuropathy, Intravenous immunoglobulin, INCAT - Overall disability sum score

## Abstract

**Background:**

Chronic immune sensory polyradiculopathy (CISP) identifies a progressive acquired peripheral dysimmune neuropathy recognized as a chronic inflammatory demyelinating polyradiculoneuropathy (CIDP) variant. We describe a young woman with a thirteen-year history of CISP with a belated variable response to intravenous immunoglobulin (IVIG) and an almost erratic anticipation of symptoms between IVIG cycles. The association of IVIG and corticosteroids, immunosuppressants, plasmapheresis, did not lead to clinical improvement and was characterized by significant side effects. We evaluated a combined clinical and somatosensory evoked potentials (SSEPs) approach aimed to identify possible predictive parameters concerning the effect and duration of each IVIG administration. Neurologic disability was evaluated using INCAT - Overall Disability Sum Score (INCAT-ODSS).

**Case presentation:**

A 30-year-old woman presented on 2004 for the subacute onset of asymmetric paresthesias in the lower limbs over the previous six months. The symptoms had been relapsing-remitting during the first four months, followed by a slow progression, resulting in limbs ataxia and a progressive gait disturbance requiring Canadian crutches. Motor and sensory nerve conduction studies and electromyographic evaluation were into normal limits. Median SSEPs were normal, while tibial SSEPs were characterised by the bilateral absence of both lumbar and cortical responses. Cerebrospinal fluid detected an increased protein concentration, while spinal MRI showed a pronounced thickening of the sacral nerve roots, together with a tube-shaped enlargement. These findings led to the diagnosis of CISP and the patient was treated with IVIG reaching a stable remission over the following 9 years. In early 2014, the patient began to show a variable response to treatment with erratic anticipation of sensory disturbances, and a more pronounced walking disability: corticosteroids, plasmapheresis, mycophenolate mofetil and cyclophosphamide were uneffective and burdened by relevant side effects. To better assess the response to IVIG in terms of time-effect, consistency and duration, we have combined a scheduled clinical and SSEPs evaluation during and after each IVIG cycle.

**Conclusions:**

The correlation between the neurophysiological data and the INCAT-ODSS scores has allowed the modulation of IVIG cycles with a significant reduction of the clinical fluctuations and disability. SSEPs may therefore represent an useful and recommended additional aid for the treatment schedule of this rare clinical form.

## Background

The term chronic inflammatory demyelinating polyradiculoneuropathy (CIDP) identifies a chronic-progressive acquired peripheral neuropathy. The clinical picture is characterised by sensorimotor signs and symptoms due to an inflammatory demyelinating process that is dysimmune in nature [1, 2].

The symptoms usually develop over a period of at least 8 weeks and are usually characterised by muscle weakness associated with sensory disturbances (paresthesia, dysesthesia, and hypoesthesia in some cases), moderate muscle wasting and areflexia. The weakness, distal and symmetric at onset, gradually tends to involve the proximal limb’s segments, resulting in a progressive disability in walking, climbing stairs and in all movements against gravity, while the cranial district is usually spared. Occasionally, a postural tremor may be present, usually due to muscle weakness [1, 2].

Several CIDP variants have been described and classified as “atypical forms” in the diagnostic criteria of the European Federation of Neurological Societies/Peripheral Nerve Society (EFNS/PNS) [3]. Chronic immune sensory polyradiculopathy (CISP) is an almost rare form: paresthesia, pain, numbness, and ataxia represent the main symptoms with an asymmetric distribution at onset and progression to a distal symmetric pattern [4]. Nerve conduction studies are normal and the diagnosis of a demyelinating process is revealed by prolonged somatosensory evoked potentials (SSEPs) [5–7]. We describe the results of a combined clinical (INCAT - Overall Disability Sum Score - ODSS) and neurophysiological (SSEPs) approach we adopted to assess the effect and duration of response to intravenous immunoglobulin (IVIG) treatment in a long-lasting case of CISP with belated variable response to treatment and erratic anticipation of sensory symptoms [8].

## Case presentation

An otherwise healthy 30-year-old woman presented on 2004 for the subacute onset of asymmetric paresthesias in the lower limbs over the previous six months. The symptoms had been relapsing-remitting during the first four months, followed by a slow progression that resulted in limbs ataxia and a progressive gait disturbance requiring Canadian crutches (ODSS: 4). Routine blood examinations, vitamins E and B12, folate, lipid profile, serum protein electrophoresis with immunofixation, ceruloplasmin, angiotensin-1-converting enzyme, thyroid function including anti-thyroid antibodies were normal. Laboratory research for neoplastic, rheumatic, celiac and venereal disease, as well as myelin associated glycoprotein, sulfatide and anti-peripheral nerve antibodies showed normal values.

Motor and sensory nerve conduction studies (median, ulnar, common peroneal, tibial, sural nerves) and electromyographic evaluation (extensor digitorum brevis, tibialis anterior, quadriceps femoris, first dorsal interosseous, extensor digitorum communis, deltoid, L4-L5 and D9 paraspinal muscles) were into normal limits, with the exception of a bilateral mild elongation of the tibial F-waves latencies (< 15% of the upper normal limit). Median SSEPs were normal, while tibial SSEPs were characterised by the bilateral absence of both lumbar (N22) and cortical (P40) responses.

A lumbar puncture detected clear cerebrospinal fluid (CSF) without cellularity, a normal glucose level (52 mg/dl) and an increased protein concentration of 128 mg/dl (NV < 50 mg/dl).

**Fig. 1:**
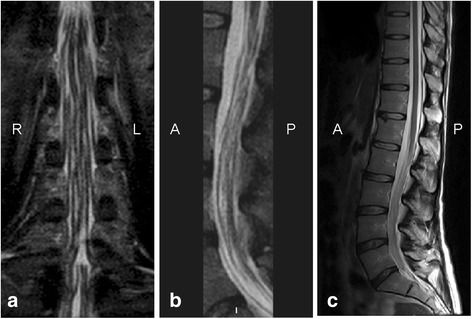
Spinal MRI study. MR coronal STIR (**a**), sagittal STIR (**b**) and T2-weighted sagittal (**c**) images of the cauda equina showing marked thickening of the nerve roots together with a tube-shaped enlargement. (R = right; L = left; A = anterior; P = posterior)

Spinal MRI showed a pronounced thickening of the sacral nerve roots, together with a tube-shaped enlargement (Fig. 1).

**Fig. 2:**
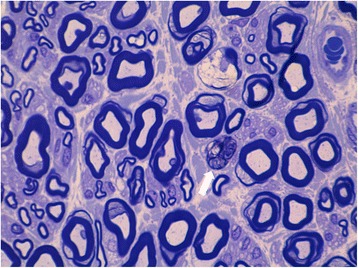
Sural nerve biopsy. Cross section of plastic embedded left sural nerve stained with Toluidin blue showing slight axonal impairment without inflammatory infiltrate, a minimal reduction in myelinated fibers density with some isolated aspects of chronic (cluster) reinnervation (white arrow) and sporadic fibers in Wallerian

A left sural nerve biopsy was performed, showing slight axonal impairment without inflammatory infiltrate, a minimal reduction in myelinated fibre density with some isolated aspects of chronic (cluster) reinnervation, and sporadic fibres in Wallerian degeneration (Fig. 2).

These findings led to the diagnosis of CISP and the patient was treated with a high dose course of methylprednisolone (500 mg/day for 8 consecutive days), without significant effects on ataxia and on walking disability (ODSS: 4–5). As a second step, ten days after high dose steroid course, she was treated with IVIG (0.4 g/kg per day, for 5 consecutive days) every 4 weeks: vibration sense and joint position began to improve after the third IVIG-dose, with a marked reduction of the ataxic pattern, and an almost normal walking ability was achieved on the tenth day (ODSS: 1–2). This regimen has ensured a prolonged and stable remission over the following 9 years with an ODSS of 0–1.

In early 2014, the patient started to have symptom fluctuations, a variable response to treatment with erratic anticipation of sensory disturbances, and a more pronounced walking disability (ODSS: 2–4): she was treated with plasmapheresis (3 a week cycles for 3 weeks without benefit), mycophenolate mofetil (500 mg per day, discontinued after 4 weeks for relevant side effects - diarrhoea and vomiting), and cyclophosphamide (100 mg per day added to IVIG, maintained for 5 months, and then discontinued because of loss of weight and leukopenia). The patient was therefore subsequently kept only on IVIG therapy. To better assess the response to IVIG in terms of time-effect, consistency and duration, we have combined a scheduled clinical (ODSS scale) and SSEPs evaluation during and after each IVIG cycle.

## Materials and methods

Basing on the patient’s IVIG regimen (5 consecutive days every 4 weeks) we have assessed bilaterally tibial and median SSEPs together with nerve conduction studies (NCS) at the first day of each IVIG administration and after 7, 14 and 21 days for 16 consecutive weeks; the neurologic disability was recorded daily using the INCAT-ODSS [8].

Obtained the informed consent, NCS and SSEPs have been performed at a skin temperature of 32 °C, following the standard guidelines [9–11].

For each registration, we have considered the “peak latency” (ms) and “peak-to-peak amplitude” (μV) for N9, N13, N20, N22, and P40 responses, with any faulty recordings being marked as “0”.

All collected data underwent statistical analysis, conducted with Statistical Package for Social Science -Version 19.0 (SPSS Inc), by setting the statistical significance level at 0.05. For descriptive statistics, we presented the data as the percentage distributions for categorical variables and as the means with standard deviations for continuous variables. Frequency distributions were compared by the chi-square test, and means were compared by the Kruskal-Wallis H-test for continuous variables. Correlations were assessed by the Spearman’s regression analysis.

## Results

**Fig. 3:**
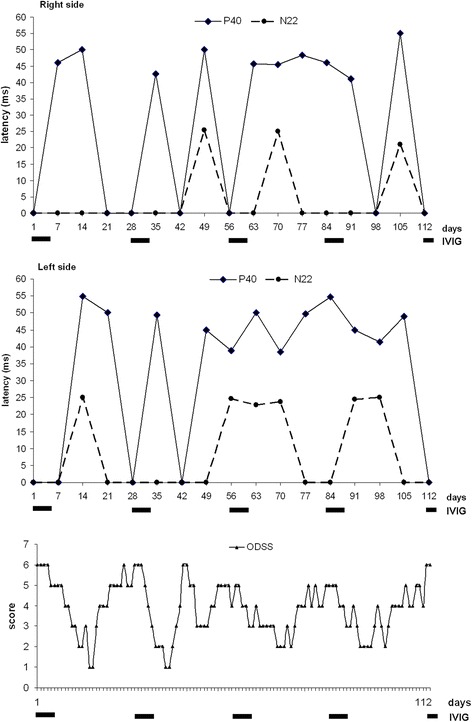
Superimposed graphs of the daily ODSS and the longitudinal trend of tibial SSEPs (N22 and P40 latencies) recorded every 7 days over a period of 16 consecutive weeks. The bold lines below the horizontal axis refer to each cycle of IVIG scheduled every 28 days. Note the large P40 latencies fluctuations significantly correlated to the highest ODSS values

**Fig. 4:**
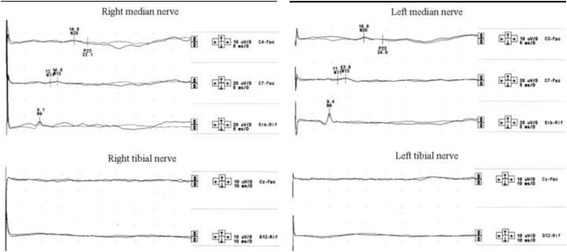
Example of SSEPs pattern recorded at the 28th-day control after an IVIG cycle. Upper traces: SSEPs from median nerve stimulation show normal responses. Lower traces: SSEPs from tibial nerve stimulation show absence of both the peripheral and cortical responses

The baseline evaluation of the SSEPs data showed a wide fluctuation of P40 latencies, ranging from 0 (41.1% of cases after right-side stimulation; 29.4% after left-side stimulation) to 55 ms for both sides (NV ≤ 44.2 ms); these values were greater than 6 standard deviations (SDs) compared with our normative data. For the N22 component, the absence of the evoked response has been documented in 82.3% of cases after right-side stimulation and in 64.7% after left-side stimulation. At the same time, ODSS values were quantified in the range of major disabilities (4–6) in 61.4% (70/114) of the total recordings, and with scores 5–6 (severe disability) in 35% (40/114) (Fig. 3-4). The amplitudes of the evoked responses (if present) were consistently 2 SDs under the normative values, with marked chronodispersion. The data derived from the upper limbs stimulation have instead shown stable and normal values for all the evoked components (N9, N13, N20), as for the central conduction time (Fig. 4).

The statistical correlation between neurophysiological data and the daily ODSS scores has shown a mean clinical worsening 16 ± 3.1 days after IVIG treatment, while the longitudinal analysis of ODSS scores, compared to the frequency of IVIG cycles (5 consecutive days every 4 weeks), has shown, for each cycles, a significant clinical worsening (ataxia) respectively at days 17, 12, 19 and 18 (Fig. 3). The isolated record on day 12 has been observed in the context of a minor infectious event of the upper airways which may contribute to a negative modulation of the dysimmune pattern. Thus excluding this confounding data, the mean clinical deterioration was estimated to occur on the eighteenth day. As a consequence, we have planned 5 consecutive IVIG doses every 18 days, repeating the neurophysiological monitoring at the first day of IVIG infusion, after 7 and 14 days for 13 consecutive registrations, together with a daily evaluation of the ODSS.

**Fig. 5:**
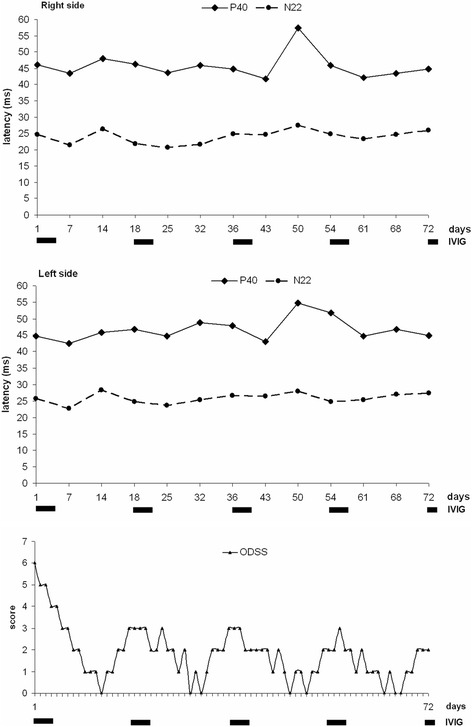
Superimposed graphs of the daily ODSS and the longitudinal trend of tibial SSEPs (N22 and P40 latencies) recorded the first day of each IVIG administration and after 7–14 days for 13 consecutive recordings. The bold lines below the horizontal axis refer to each cycle of IVIG scheduled every 18 days. Note the relevant stabilization of P40/N22 latencies and the reduction of ODSS values

**Fig. 6:**
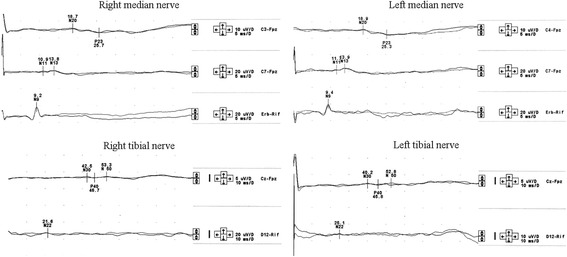
Example of SSEPs pattern recorded at the 18th-day control after an IVIG cycle. Upper traces: SSEPs from median nerve stimulation show normal responses. Lower traces: SSEPs from tibial nerve stimulation show the presence of both the peripheral (N22) and cortical (P40) response, although the latter with increased latency

The statistical analysis of the new data collection has shown, unlike the baseline assessments, a) an always reliable N22 and P40 responses; b) a significant reduction of P40 and N22 latencies (*p* < 0.04) with a mean values of 45.6 ± 3.9 ms (range: 42.0–57.0) in the right-side, 46.6 ± 3.4 ms (range: 42.4–54.7) in the left side for P40, and 24.0 ± 2.0 ms (range: 20.7–27.4) in the right side, 25.6 ± 1.58 ms (range: 22.8–28.4) in the left side for N22, respectively. Note that the highest scores (57 ms for P40, and 27.4 ms for N22) have been recorded only once during the whole neurophysiological monitoring (Figs. 5 and 6). The ODSS scores were in the range 0–2 (moderate or absent disability) in 77.7% (56/72) of the total recordings, and with score 3 (“requires unilateral support to walk 10 meters - stick, single crutch, one arm”) in only 15.2% (11/72) (Fig. 5). When compared to baseline results, the new data are much more homogeneous, as confirmed by the significant reduction of SDs from the average values (1–1.5 SDs).

The time course analysis of P40 latencies (from the first day of IVIG infusion to the beginning of the subsequent cycle) showed an improving trend, for the right side, between the first and the second week (from a mean value of 45.5 ± 0.71 ms to 42.7 ± 0.9 ms), followed by a worsening trend between the second and third week (mean value: 48,65 ± 6,06 ms). These trends were found to be statistically significant (*p* = 0.046 - Kruskal-Wallis H test), and similar results have been detected on the left side, reaching the threshold of statistical significance (*p* = 0.051). The pejorative and ameliorative trend is also confirmed by the changes in ODSS scores, from a mean value of 2.6 in the first week (discrete disability), to 0.8 in the second week (minimal disability), and finally equal to 1.8 in the third week (*p* < 0.001 - Kruskal-Wallis H test). No statistical correlation has been found considering N22 and P40 amplitudes.

Nerve conduction studies were consistently within the normative values, including F-waves, without statistical correlation between clinical exacerbations or improvement after each IVIG protocol.

## Discussion and conclusion

An inflammatory radiculoneuropathy with the predominant involvement of the dorsal roots has been originally described by Sinnreich as a cause of sensory ataxia, introducing the term of chronic immune sensory polyradiculopathy (CISP). All the patients had gait ataxia, large-fibre sensory impairment, paraesthesias, an high CSF protein level, a completely normal motor and sensory nerve conduction studies, and abnormal SSEPs as a specific hallmark, as in our case [12–15]. Moreover, the neuropathological findings were also not specific for a peripheral demyelinating process, and ruled out the presence of an inflammatory infiltrate [16].

According to the EFNS/PNS guidelines CISP is clinically classified within the subgroup of atypical-CIDP, but the diagnosis is feasible only by considering several “supportive criteria”, and it cannot be further characterised in terms of “definite”, “probable” or “possible” since electrodiagnostic criteria do not include SSEPs, which represent the more sensitive diagnostic tool [3, 17]. Several authors have indeed recently underlined the diagnostic properties and utility of SSEPs in evaluating CIDP patients [18–21].

In the present case, considering clinical fluctuations and the erratic response to IVIG treatment, we have planned a neurophysiological approach (SSEPs) together with the daily ODSS record, not only for a diagnostic confirmation, but also to search possible correlations between neurophysiological and clinical data potentially exploitable for therapeutic purposes. Consider also that we often modulate “empirically” the IVIG cycles following the criterion of the clinical worsening, as well as the choice of other therapeutic measures (corticosteroids, immunosuppressants, plasmapheresis) [22–24].

Our data, although derived from a single case, show a close correlation between serial SSEPs and ODSS scores, thus contributing to the assessment of the effect and duration of IVIG administration and, accordingly, into the modulation of the frequency of IVIG intake, allowing the patient to reduce both clinical fluctuations and disability with any further significant relapse. In addition, the new therapeutic regimen, scheduled according to the neurophysiological evidences, has enabled a significant improvement of the ODSS global average score from 3.85 to 1.83.

We agree with some previous evidences about the non-localizing property of tibial SSEPs in the demyelinating process but, as shown in this case, they may be an useful supportive aid to establish the more appropriate therapeutic program faithfully reproducing the evolution of the clinical picture [4, 18, 21]. Undoubtedly, none of the two resources (SSEPs and ODSS) reaches an absolute diagnostic power, but we recommend their execution and integration in such rare and selective clinical forms.

## Abbreviations

CIDP: Chronic inflammatory demyelinating polyradiculoneuropathy
CISP: Chronic immune sensory polyradiculopathy
CSF: Cerebrospinal fluid
EFNS/PNS: European Federation of Neurological Societies/Peripheral Nerve Society
INCAT-ODSS: Inflammatory Neuropathy Cause and Treatment - Overall Disability Sum Score
IVIG: Intravenous immunoglobulin
MRI: Magnetic resonance imaging
NV: Normal value
SDs: Standard Deviations
SPSS: Statistical Package for Social Science
SSEPs: Somatosensory evoked potentials
